# Cell adhesion to the extracellular matrix protein fibronectin modulates radiation-dependent G2 phase arrest involving integrin-linked kinase (ILK) and glycogen synthase kinase-3***β*** (GSK-3***β***) *in vitro*

**DOI:** 10.1038/sj.bjc.6600912

**Published:** 2003-04-29

**Authors:** N Cordes, D van Beuningen

**Affiliations:** 1 Institute of Radiobiology, German Armed Forces Medical Academy, Neuherbergstrasse 11, 80937 Munich, Germany

**Keywords:** ECM, radiosensitivity, ILK, GSK-3*β*, cell cycle, G2 arrest

## Abstract

Cell adhesion to extracellular matrix (ECM) is thought to confer resistance against cell-damaging agents, that is, drugs and radiation, in tumour and normal cells *in vitro*. The dependence of cell survival on *β*1-integrin-linked kinase (ILK), protein kinase B*α*/Akt (PKB*α*/Akt) and glycogen synthase kinase-3*β* (GSK-3*β*) activity, which participate in *β*1-integrin signalling and cell cycle progression was investigated as a function of radiation exposure. Colony-formation assays on polystyrene, fibronectin (FN), laminin (LA), bovine serum albumin (BSA) or poly-L-lysine (poly-L) (0–8 Gy), kinase assays, flow cytometric DNA and annexin-V analysis and immunoblotting were performed in nonirradiated and irradiated (2 or 6 Gy) A549 human lung cancer cells and CCD32 normal human lung fibroblasts. Cell contact to FN in contrast to polystyrene elevated basal ILK, PKB*α*/Akt and GSK-3*β* kinase activities in A549 and CCD32 cells, as well as the basal amount of A549 G2 phase cells. Irradiation on FN or LA as compared to polystyrene, BSA or poly-L significantly improved cell survival. Following irradiation, kinase activities were stimulated strongly on polystyrene but showed to be less prominent on FN, which was because of the FN-related basal induction. Following irradiation, FN compared to polystyrene enlarged and prolonged G2 arrest in both the cell lines. For the analysis of phosphatidylinositol-3 kinase (PI3-K) dependence of protein kinases and cell cycle transition, the PI3-K inhibitors LY294002 and wortmannin were used showing decreased kinase activities, antiproliferative and radiation-dependent G2 accumulation-abrogating effects accompanied by downregulation of cyclin D1 and phospho-pRb in cells attached to polystyrene. Fibronectin partly abrogated these effects PI3-K-independently. These findings suggest a novel pathway that makes direct phosphorylation of GSK-3*β* by ILK feasible after irradiation. Conclusively, the data indicate that ILK, PKB*α*/Akt and GSK-3*β* are involved in modulations of the cell cycle after irradiation. These interactions are strictly dependent on ECM components in a cell line-specific manner. Our findings provide molecular insights into mechanisms likely to be important for ECM-dependent cell survival and cellular radioresistance as well as tumour growth.

The presence of extracellular matrix (ECM) increases cellular resistance to cell-damaging agents such as drugs or ionising radiation (Damiano *et al*, 1999; Rose *et al*, 1999; Hazlehurst *et al*, 2000; Cordes *et al*, 2002; Cordes and Meineke, in press; Cordes *et al*, in press). For the treatment of cells with cytotoxic agents in the presence of ECM, the convergence of integrin-related and cell cycle-related signal transduction pathways could be of great importance. Possible interactions of these pathways are likely to be modulated by the ECM and, thus, may play an important role in resistance-mediating mechanisms (Sethi *et al*, 1999; Hazlehurst *et al*, 2000), anchorage-independent cell growth (Shin *et al*, 1975; Radeva *et al*, 1997), oncogenic transformation (White *et al*, 2001), the assessment of *in vitro* cytotoxicity studies, and new strategies for controlling and healing cancer.

Signals generated by cell–ECM contact to critical cellular processes, that is, cell cycle transition, survival, differentiation, migration or adhesion, are transmitted in particular by the integrin receptor family (Hynes, 1992; Giancotti and Ruoslahti, 1999). Among at least 22 different *α*/*β*-heterodimers, the *β*1-integrin is the most widespread receptor subunit existing in combination, for example, with *α*5 the fibronectin receptor (Argraves *et al*, 1987) and with *α*1, *α*3 or *α*6 receptors for laminin or collagen (Hynes, 1992). Initiated integrin clustering after cell–ECM attachment activates intracellular effectors that are capable of coupling integrins and growth factor receptors to specific downstream targets such as *β*1-integrin-linked kinase (ILK) (Hannigan *et al*, 1996) or focal adhesion kinase (Schaller and Parsons, 1994). *β*1-integrin-linked kinase functioning in a phosphatidylinositol-3 kinase (PI3-K)-dependent manner (Delcommenne *et al*, 1998) is able to stimulate protein kinase B*α*/Akt (PKB*α*/Akt) and glycogen synthase kinase-3*β* (GSK-3*β*) (Troussard *et al*, 1999). These events proceed in inhibition of apoptosis by phosphorylating and inactivating apoptotic factors such as caspase-9 and Bad through PKB*α*/Akt (Datta *et al*, 1997; Khwaja *et al*, 1997; Tian *et al*, 2002) and cell cycle progression via prevention of cyclin D1 proteolysis (Diehl *et al*, 1998) and gene expression via AP-1 or NF-*κ*B through GSK-3*β* (Troussard *et al*, 1999; Hoeflich *et al*, 2000).

Concerning the coactivation of in-membrane complexes-associated integrin and growth factor receptors, identical intracellular biochemical pathways are used by the different receptors, but in many cases they do it at different steps (Pawson and Saxton, 1999; Schwartz and Baron, 1999). These coactivations are thought to be necessary in certain cases for optimal signal transduction to regulate diverse cellular functions (Schwartz and Ginsberg, 2002; Yamada and Even-Ram, 2002). Thus, the treatment of ECM-attached cells with external cytotoxic stimuli is likely to show a modulated responsive pattern in comparison with artificial substrates, that is, polystyrene (Meredith *et al*, 1993; Giancotti and Ruoslahti, 1999).

An integral part of the cellular radiation response pattern upon DNA damage is the blockage of the cell cycle. Recent findings also reported intense alterations of cell cycle regulatory proteins through cell detachment presenting similar radiation-related DNA damages (Kang and Krauss, 1996; Zhu *et al*, 1996; Wu and Schönthal, 1997). The question that still remains unclear is what are the acute consequences on the cell cycle after irradiation of monolayer cultures in an optimised *in vitro* microenvironment.

To elucidate the molecular mechanisms involved in improved clonogenic cell survival after irradiation by ECM proteins, we analysed the acute changes of the activities of the upstream-located *β*1-integrin signalling pathway protein kinases ILK, PKB*α*/Akt and GSK-3*β*, as well as cell cycle progression in human lung tumour cells and normal human lung fibroblasts *in vitro*.

## MATERIALS AND METHODS

### Cells

The lung carcinoma cell line A549 was purchased from the American Type Culture Collection (ATCC, Rockville, MD, USA) and the normal human lung fibroblastic stem line (CCD32) was a generous gift from Professor HP Rodemann (Section of Radiobiology and Molecular Environmental Research, University Tuebingen). Dulbecco's modified Eagle's medium (DMEM) (PAA, Linz, Austria) supplemented with 1% nonessential amino acids and 10% foetal bovine serum (FBS; PAA, Linz, Austria) was applied to culture the cells. Routinely, cells were incubated at 37°C in a humidified atmosphere containing 10% CO_2_ buffered at pH 7.35. Where indicated, serum starvation of cells was always performed using DMEM supplemented with 1% nonessential amino acids without FBS. All experiments using CCD32 cells were performed between passages 5 and 15. Furthermore, for all experiments only asynchronous, exponentially growing cell cultures were employed.

### Radiation exposure

Irradiation was delivered at room temperature using single doses of 240 kV X-rays (Isovolt 320/10; Seifert, Ahrensburg, Germany) filtered with 3 mm Be. The absorbed dose was measured using a Duplex dosimeter (PTW, Freiburg, Germany). The dose rate was approximately 1 Gy min^−1^ at 13 mA. The applied doses ranged from 0 to 8 Gy.

### Colony-formation assay

The colony-formation assay was applied for the measurement of clonogenic cell survival. Exponentially growing A549 or CCD32 cells were plated onto noncoated or fibronectin (FN; 1 *μ*g cm^−2^; Becton Dickinson, Heidelberg, Germany), laminin (LA; 1 *μ*g cm^−2^; Sigma-Aldrich GmbH, Taufkirchen, Germany), bovine serum albumin (BSA; 1 *μ*g cm^−2^; GIBCO, Karlsruhe, Germany) or poly-L-lysin (poly-L; 1 *μ*g cm^−2^; Calbiochem-Novabiochem GmbH, Bad Soden, Germany) precoated six-well dishes (Becton Dickinson, Heidelberg, Germany) 36 h prior to irradiation. To examine the effect of the specific PI3-K inhibitor LY294002 (Sigma-Aldrich GmbH, Taufkirchen, Germany) on clonogenic survival, cells were seeded on polystyrene or FN for 24 h, serum-starved and incubated with concentrations of 0.1–100 *μ*M of the inhibitor for 18 h. In combination with irradiation, cells were prepared as described and withdrawal of the inhibitor was performed immediately prior to radiation. At 8–10 days after irradiation, grown colonies were stained with Coomassie blue. Colonies greater than 50 cells were counted. All experiments were repeated three times.

### *β*1-integrin-linked kinase assay and Western blotting

As reported, ILK (Delcommenne *et al*, 1998) and PKB*α*/Akt (Cross *et al*, 1995) are able to phosphorylate GSK-3*β in vitro*. Cells were plated onto polystyrene or FN for 24 h, subsequently serum-starvation ±50 *μ*M LY294002 or 100 nM wortmannin occurred for 18 h prior to irradiation (6 Gy). Cell lysis subsequent to irradiation was performed for 10 min on ice at 5 and 60 min. Untreated and treated cells were scrapped off and 500 *μ*g of the total protein extracts were incubated for 3 h, firstly, with monoclonal ILK antibodies clone 65.1.9 (Upstate, Hamburg, Germany) at 4°C and, secondary, with protein-A–agarose beads (Sigma-Aldrich GmbH, Taufkirchen, Germany) at room temperature. Immunoprecipitated ILK was then employed for protein kinase activity measurements using kinase buffer plus GSK-fusion protein and 200 *μ*M ATP (New England Biolabs, GmbH, Frankfurt a.M., Germany). After a 30 min incubation at 30°C, the reaction was terminated with 4 × SDS-sample buffer. Immunoprecipitates were separated by SDS–polyacrylamide electrophoresis, transferred onto a nitrocellulose membrane (Schleicher and Schuell GmbH, Dassel, Germany), blocked using 5% nonfat dry milk powder in PBS and probed overnight at 4°C with rabbit antiphospho-GSK-3*β* antibodies specific for Ser9 (New England Biolabs, GmbH, Frankfurt a.M., Germany). Three independent experiments were performed. The protein detection was accomplished using specific HRP-conjugated goat anti-rabbit antibodies in combination with the enhanced chemiluminescence detection systems (ECL; Amersham, Freiburg, Germany). Measurements of protein band density were carried out using ImageQuant version 5.0 software (Molecular Dynamics, Krefeld, Germany).

### PKB*α*/Akt kinase assay

The nonradioactive Akt kinase assay kit purchased from New England Biolabs, GmbH, Frankfurt a.M., Germany, was used. Cells were plated onto polystyrene or FN for 24 h, subsequently serum-starvation ±50 *μ*M LY294002 or 100 nM wortmannin occurred for 18 h prior to irradiation (6 Gy). Then, cells were processed as recommended by the manufacturer's device. Immobilised Akt antibody agarose beads were used for immunoprecipitation. Subsequently, kinase buffer, 200 *μ*M ATP and GSK-fusion protein were added to the PKB–agarose bead complex. After a 30 min incubation at 30°C, the reaction was terminated and SDS–polyacrylamide electrophoresis and Western blotting were performed as described above. Experiments were repeated three times.

### Total protein extractions and antibodies

Polystyrene- or FN-grown cells were irradiated with 6 Gy or left untreated. For protein extraction for GSK-3*β*, nonirradiated, irradiated ±LY294002 (50 *μ*M)- or wortmannin (100 nM)-treated cells were lysed at 5 and 60 min using 50 mM Tris-HCl (pH 7.4), 1% NP-40, 0.25% sodium deoxycholate, 150 mM NaCl, 1 mM EDTA supplemented with protease inhibitor cocktail complete® (Roche Diagnostics GmbH, Mannheim, Germany), 5 mM sodium vanadate and 5 mM sodium fluoride. Cell homogenisation using a 25-gauge needle was followed by a 30 min incubation on ice. For protein extraction for cell cycle proteins, cells grown on FN or polystyrene were exposed to 6 Gy, 50 *μ*M LY294002 or left untreated. At 2, 6, 12 and 24 h after irradiation or a 12-h LY294002 incubation, cells were lysed using 50 mM HEPES, 250 mM sucrose, 1 mM EDTA, 5 mM MgSO_4_, 1 mM dithiothreitol, 5 mM sodium vanadate, 5 mM sodium fluoride and protease inhibitor cocktail complete®. A 5-min incubation on ice was followed by freezing three times in liquid nitrogen and thawing at 37°C. Amounts of total protein extracts were determined using a spectral-photometer (helios alpha, Unicam instruments, Cambridge, UK) and stored at −134°C until use. Experiments were repeated three times. Western blotting was performed as described above using 25 *μ*g of total protein extracts. Anti-GSK-3*β* (1 : 2000), antiphospho-GSK-3*β*-Ser9 (1 : 1000), antiphospho-pRb-Ser795 (1 : 500) (New England Biolabs GmbH, Frankfurt a.M., Germany), antiretinoblastomaprotein (pRb) (1 : 1000; Santa Cruz, Heidelberg, Germany), anti-PKB*α*/Akt (1 : 1000), anticyclin D1 (1 : 1000) (Becton Dickinson, Heidelberg, Germany) and anti-*β*-actin (1 : 5000; Sigma-Aldrich GmbH, Taufkirchen, Germany) antibodies and specific HRP-conjugated goat anti-rabbit and anti-mouse secondary antibodies (Santa Cruz, Heidelberg, Germany) were used.

### Flow cytometric analysis of apoptosis by annexin-V staining

The effect of LY294002 on the induction of apoptosis was analysed by flow cytometry. Treatment of 1 × 10^6^ cells grown on polystyrene or FN with 50 *μ*M LY294002 for 18 h in combination with serum deprivation was followed by detachment of cells using trypsin/EDTA, washing with PBS and preparation for analysis following the instructions of the annexin-V-FLUOS staining Kit (Roche, Germany). After simultaneous staining of cells with annexin-V—fluorescein plus propidium iodide solution for 15 min, acquisition of data for 10 000 events was performed using a Becton Dickinson Fluorescence-activated Cell Sorter (FACScan). The distribution and differentiation of vital, apoptotic and necrotic cells were analysed from the dot plots using CELLQuest software. Experiments were repeated two times.

### Cell cycle analysis

Nonirradiated or irradiated cells (1 × 10^6^) grown on FN or polystyrene were detached at 0, 4, 8, 12 and 24 h, washed twice with PBS and fixed in 80% ethanol until use. Where indicated, cells were incubated with 50 *μ*M LY294002 and serum starved from 18 h prior to irradiation up to 24 h after irradiation. Cells were prepared for analysis following the instructions of the CycleTEST™ PLUS DNA Reagent Kit (Becton Dickinson, Heidelberg, Germany). After staining of cells with propidium iodide solution for 30 min, acquisition of data for 10 000 events was performed with the means of a FACScan. The distribution of cells in the different phases of the cell cycle was analysed from the DNA-histograms using CELLQuest software. Experiments were repeated three times.

### Data analysis

Means±s.d. of surviving fractions, protein kinase activities, induction of apoptosis and cell cycle data were calculated with reference to untreated controls defined as 1.0 or in a percentage scale. To test statistical significance, analysis of variance was performed by means of ANOVA with a software package (Microsoft, Exel 97) on IBM computer systems. Results were considered statistically significant if a *P*-value of less than 0.05 was reached. The fit of the dose–effect curves was calculated by means of the linear-quadratic model (log *S*=−*αD*−*βD*^2^).

## RESULTS

### Extracellular matrix-dependent colony formation

**Figure 1:**
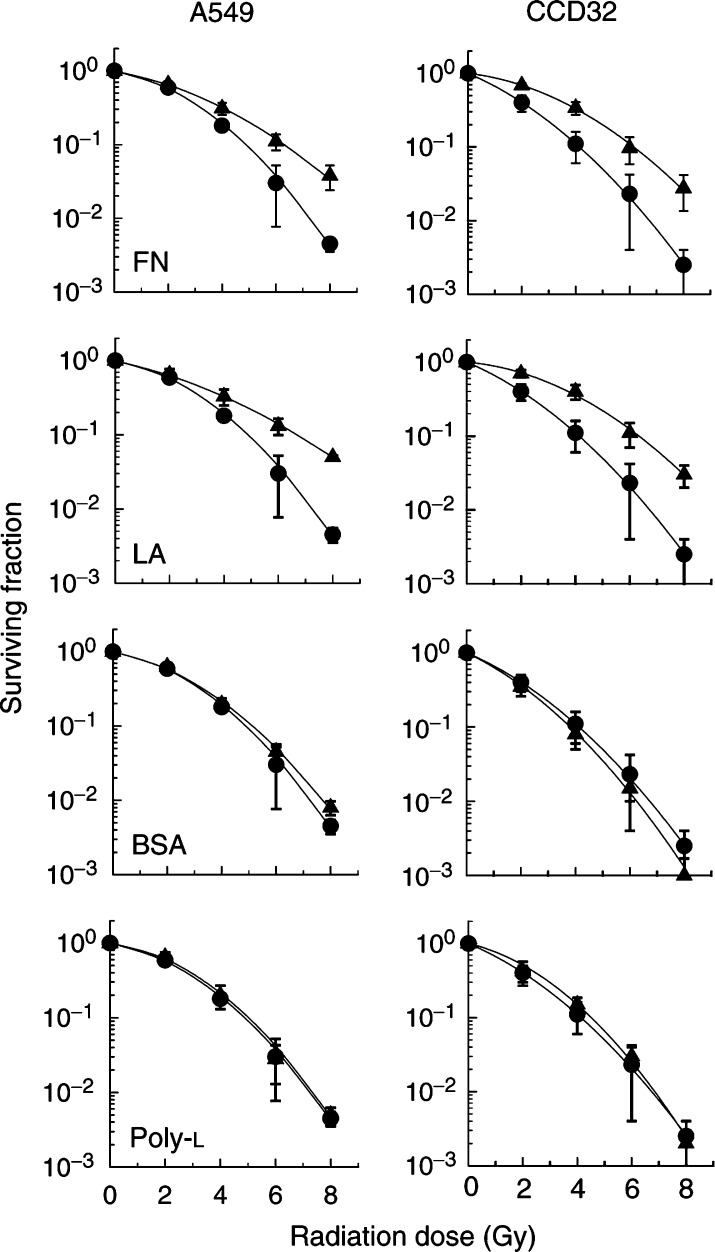
Analysis of substrate-dependent clonogenic cell survival was accomplished by plating human lung carcinoma cells A549 and normal human lung fibroblasts CCD32 24 h prior to irradiation onto polystyrene (•) or a specific protein (▴: FN, LA, BSA, poly-L). *P*<0.05 was found for radiation doses ⩾4 Gy for FN- or LA-attached A549 cells and ⩾2 Gy for FN- or LA-attached CCD32 cells, compared to polystyrene, BSA or poly-L. Each data point represents the mean±s.d. of three independent experiments (*n*=18).

To test whether FN or LA influences the clonogenic survival of A549 and CCD32 cells after irradiation, cells were plated onto FN-, LA-, BSA- or poly-L-coated or noncoated surfaces. The plating efficiency of cells was ∼70% (A549) or ∼10% (CCD32) independent of the substrate on which the cells were plated. Significantly improved survival could be detected for irradiated cells grown on FN or LA as compared to culture polystyrene, BSA or poly-L (Figure 1

). The *P*-values of radiation doses ⩾4 Gy for A549 cells on FN or LA and ⩾2 Gy for CCD32 cells on FN or LA were calculated to be less than 0.05.

### *β*1-integrin-linked kinase, PKB*α*/Akt and GSK-3*β* kinase activities

**Figure 2:**
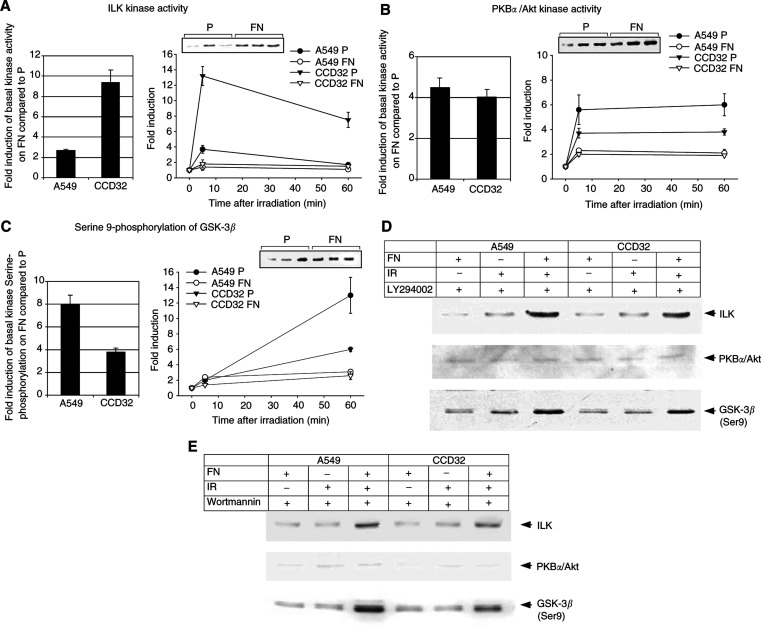
Protein kinase activities of ILK (**A**) and PKB*α*/Akt (**B**) and GSK-3*β* phosphorylation at the amino-acid residue Ser9 (**C**) were examined in A549 and CCD32 cells attached to polystyrene (P) or FN at 5 or 60 min after irradiation with 6 Gy (right panels). Basal kinase activities were strongly stimulated by FN in both tumour and normal cells (left panels). Radiation-dependent increases of ILK and PKB*α*/Akt activity and GSK-3*β* phosphorylation demonstrated to be pronounced on polystyrene and less prominent on FN. Additionally, ILK, PKB*α*/Akt and GSK-3*β* protein were detected to exclude changes in total protein amounts and *β*-actin served as loading control (data not shown). Each data point shown represents the fold induction (means±s.d.) of protein kinase activity or Ser9 phosphorylation of GSK-3*β* from the densitometric protein band analysis of three independent experiments in relation to untreated controls (mock). Inset, photographic demonstration of one exemplary protein kinase assay used for densitometric analysis. To examine the dependence of ILK, PKB*α*/Akt activity and GSK-3*β* phosphorylation on the PI3-K pathway, cells attached to FN or polystyrene were incubated with the PI3-K-specific inhibitors LY294002 (50 *μ*M) (**D**) or wortmannin (100 nM) (**E**) in serum-free medium and then irradiated with 6 Gy (IR). The data uncovered PI3-K-dependent decrease of basal and radiation-induced kinase activities (ILK and PKB*α*/Akt at 5 min; GSK-3*β* at 60 min) in cells grown on FN or polystyrene. However, irradiation on FN was able to stimulate ILK and GSK-3*β* independent of PI3-K indicating the involvement of yet unknown signalling pathways.

We analysed ILK, PKB*α*/Akt and GSK-3*β* activities to evaluate their role within the acute cellular radiation response (Figure 2A–C

).

Concerning ILK, we could show a fast and transient radiation-dependent induction in both A549 and CCD32 cells (Figure 2A). Radiation-induced ILK activity in cells grown on FN demonstrated to be less prominent compared to cells attached to polystyrene. This difference in induction might be because of cell adhesion-mediated elevation of basal ILK activity on FN compared to polystyrene. Additionally, PKB*α*/Akt and GSK-3*β* also demonstrated elevated basal kinase activity or Ser9 phosphorylation, respectively (Figure 2A–C). The downstream target of ILK PKB*α*/Akt showed a fast activation within 5 min after irradiation (Figure 2B). Subsequently, in contrast to ILK, PKB*α*/Akt remained on a high level up to 60 min post irradiation. For GSK-3*β*, we detected a strong increase in phosphorylation at the amino-acid residue Ser9 by irradiation in cells grown on FN or polystyrene (Figure 2C).

To investigate PI3-K-dependence of ILK, PKB*α*/Akt and GSK-3*β* (Delcommenne *et al*, 1998), cells were exposed to 50 *μ*M LY294002 or 100 nM wortmannin. Both A549 and CCD32 cells grown on polystyrene or FN demonstrated pronounced decreases in basal ILK and PKB*α*/Akt activity as well as GSK-3*β* phosphorylation after incubation with these inhibitors indicating a PI3-K-dependent mechanism (Figure 2D and E). Most interestingly, in contrast to PKB*α*/Akt, the inhibitory effect of LY294002 or wortmannin was partly abolished by irradiation in ILK and GSK-3*β* when cells had FN contact.

### Dependence of colony formation and induction of apoptosis on PI3-K

**Figure 3:**
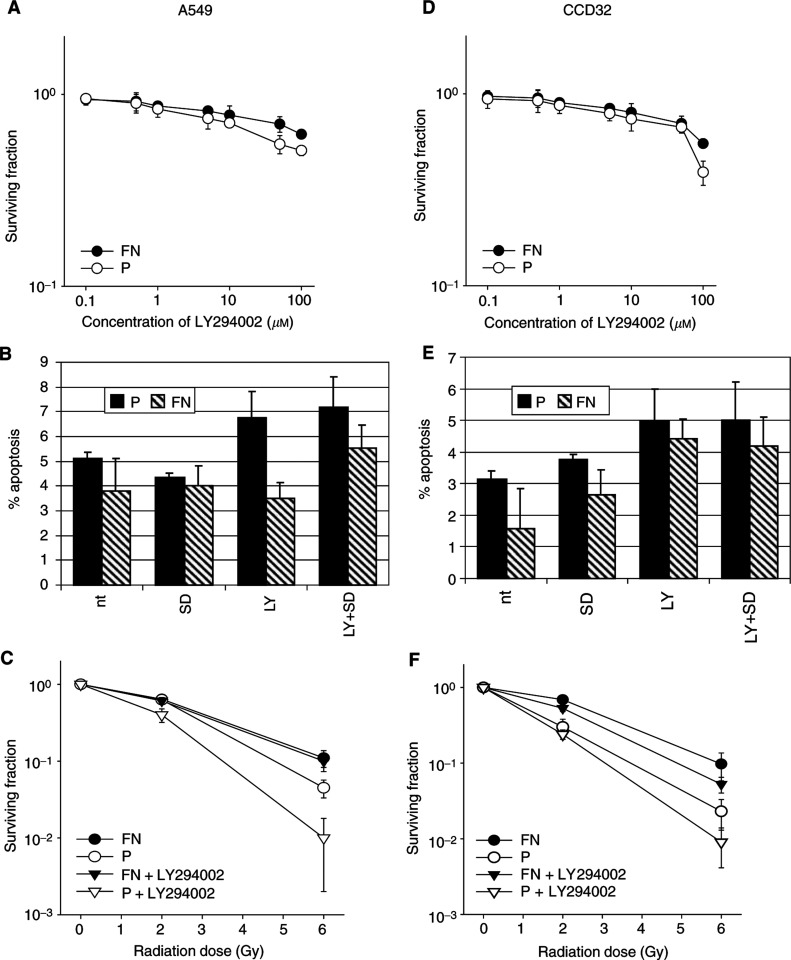
(**A**–**F**) An 18-h inhibition of PI3-K by LY294002 using concentrations from 0.1 to 100 *μ*M in combination with serum starvation resulted in a nonlinear and polystyrene (P)- or FN-independent dose–response relation for A549 (**A**) and CCD32 cells (**D**). Determination of apoptosis of A549 (**B**) or CCD32 cells (**E**) induced by LY294002 (LY) plus serum starvation (SD) on polystyrene or FN in comparison with untreated controls (nt) was performed by annexin-V staining and flow cytometric analysis. Cell viability was >90% in all the cases. The radiation-dependent clonogenic cell survival of A549 (**C**) and CCD32 cells (**F**) grown on FN or polystyrene was tested after an 18-h incubation with the PI3-K inhibitor LY294002 (50 *μ*M) in serum-free medium. The data indicate no significant change of the clonogenic survival of irradiated cells on FN, whereas on polystyrene the survival was further reduced by LY294002 at 2 and 6 Gy in A549 cells and at 6 Gy in normal lung fibroblasts significantly (*P*<0.05).

Single 18-h LY294002 treatment using various concentrations in combination with serumdeprivation resulted in a nonlinear and matrix-independent dose–response relation for A549 and CCD32 cells (Figure 3A and D

). Choosing a 50 *μ*M LY294002 concentration for further experiments was based on an acute, noncytotoxic effect of the agent, which was determined by measurement of apoptosis using annexin-V staining (Figure 3B and E), as well as a strong inhibition of PI3-K-dependent protein kinases ILK, PKB*α*/Akt and GSK-3*β*. Investigating the influence of PI3-K inhibition in combination with irradiation on the clonogenic survival of A549 and CCD32 cells was performed to assess the consequences seen in the acute alterations of ILK, PKB*α*/Akt and GSK-3*β* (Figure 3C and F). Fibronectin counteracted significantly (*P*<0.01) the cytotoxic effect of LY294002 in both irradiated A549 and CCD32 cells.

### Cell cycle analysis

**Figure 4:**
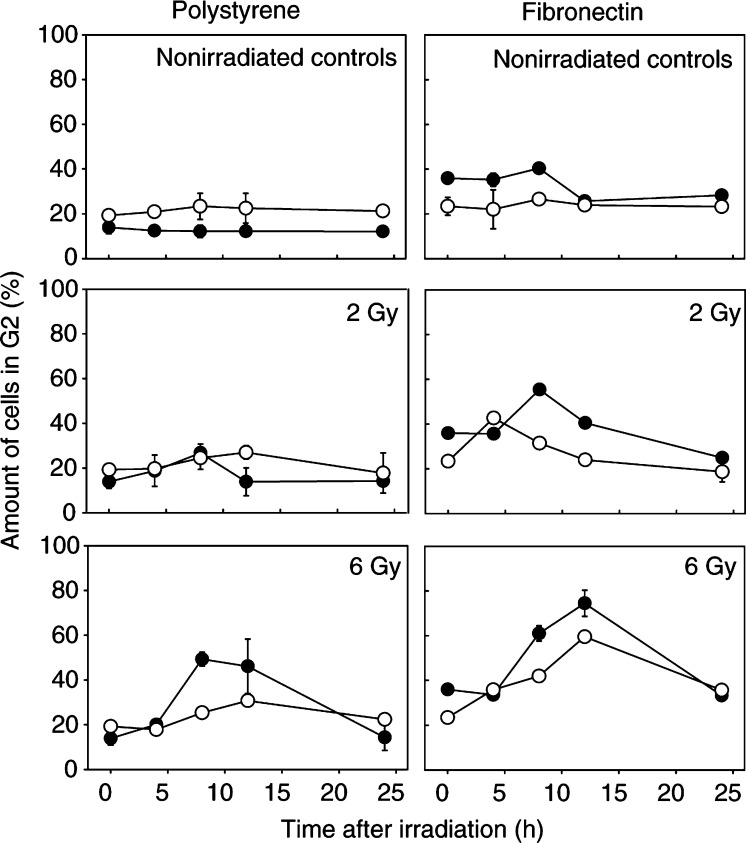
G2 phase distribution of nonirradiated or irradiated (2 or 6 Gy) A549 (•) and CCD32 cells (○) grown on polystyrene or FN is shown. Each data point represents the mean ± s.d. of three independent experiments. A549 cells, but not CCD32 cells, indicated an elevated basal amount of G2 cells at FN presence. Fibronectin prolonged and increased the radiation-induced G2 phase blockage in both the cell lines in a dose-dependent manner.

Next, the influence of FN on the cell cycle response of irradiated A549 and CCD32 cells was examined (Figure 4

) with regard to ILK and GSK-3*β* involvement in the regulation of cell cycle events (Radeva *et al*, 1997). As indicated by flow cytometry, nonirradiated A549 cells grown on FN demonstrated an increase of G2 phase cells by a two-fold and a concomitant decrease of S phase cells 24 h after plating in comparison with cells plated onto polystyrene. In contrast, nonirradiated CCD32 cells grown on FN or polystyrene did not show any differences in cell cycle distributions.

A549 cells on FN presented a dose-dependent G2 block, which showed to be increased and prolonged compared to the G2 phase block seen in cells on polystyrene (Figure 4). Cells attached to FN accumulated significantly (*P*<0.01) in G2 from 36% in nonirradiated controls to 56% (2 Gy) or 75% (6 Gy) and on polystyrene from 14 to 27% (2 Gy) or 50% (6 Gy) (Figure 4). Cells in the G0/G1 or S phase shifted concomitantly to the changes detected in G2. Concerning CCD32 cells, irradiation induced a dose-dependent G2 phase arrest. Similar to A549 cells, FN markedly altered the cell cycle distribution presenting 43 or 60% G2 phase cells after 2 or 6 Gy compared to 23% in the untreated control (Figure 4). Irradiation on polystyrene resulted in an accumulation of G2 phase cells from 19% in the untreated control to 27% (2 Gy) or 30% (6 Gy) (Figure 4). Cells in the G0/G1 or S phase shifted concomitantly to changes detected in G2. In both A549 and CCD32 cells, the normal cell cycle distribution similar to untreated controls was reached 24 h after irradiation.

**Figure 5:**
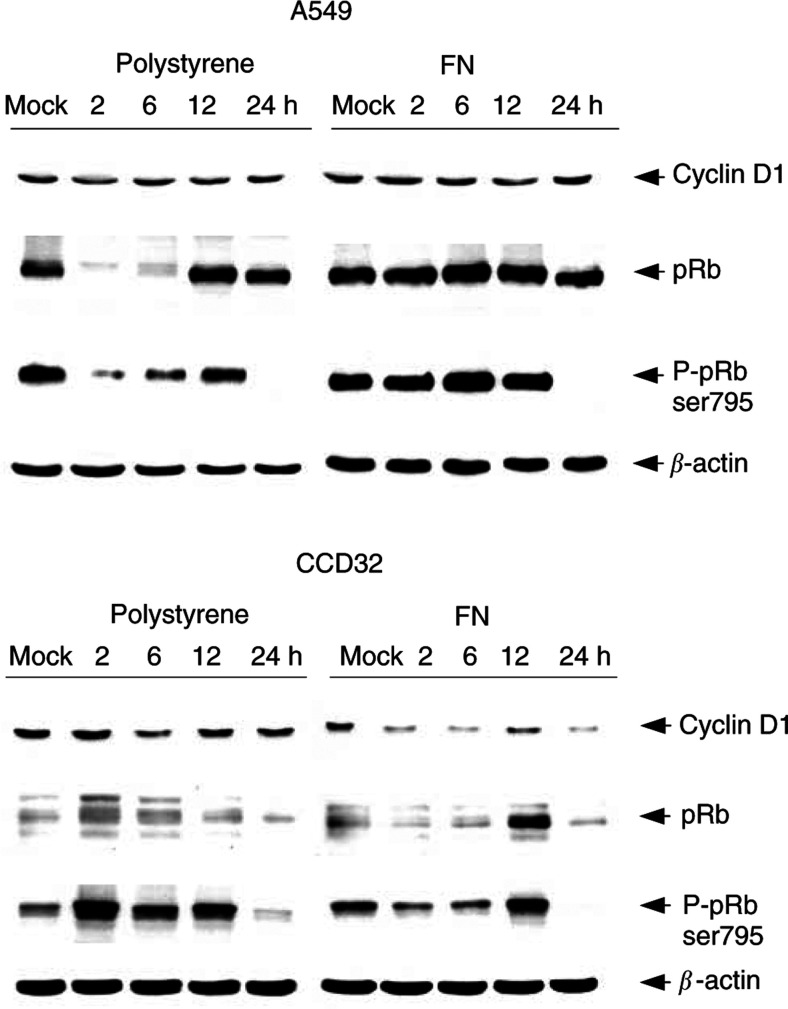
Changes in the expression of indicated cell cycle proteins in nonirradiated (mock) or irradiated (6 Gy) A549 and CCD32 cells grown on polystyrene or FN are shown. Protein extracts of irradiated cells were isolated 2, 6, 12 and 24 h postradiation exposure. Detection of *β*-actin is shown to confirm equal loading of all lanes.

In spite of G2 phase arrest in both the cell lines on polystyrene and FN observed by flow cytometry (see Figure 4), A549 and CCD32 cells showed pronounced differences in cyclin D1, pRb and phospho-pRb expression patterns comparing polystyrene to FN (Figure 5A and B

). A549 cells grown on both FN and polystyrene demonstrated decreases in cyclin D1 at 2 h on polystyrene and at 12 h on FN. In parallel, pRb protein and Ser795 phosphorylation diminished almost completely after irradiation on polystyrene, whereas on FN the strong Ser795 phosphorylation remained unchanged. At 24 h postirradiation, pRb Ser795 phosphorylation was undetectable on both the substrates (Figure 5A).

Concerning CCD32 cells, cyclin D1 decreased at 6 h on polystyrene and continuously on FN (Figure 5B). The pattern of pRb protein and pRb phosphorylation showed similarity to A549 cells with regard to the dramatic reduction at 24 h postirradiation. On polystyrene, pRb expression was induced after irradiation and reduced on FN. The phosphorylation of pRb at Ser795 was changed in parallel.

**Figure 6:**
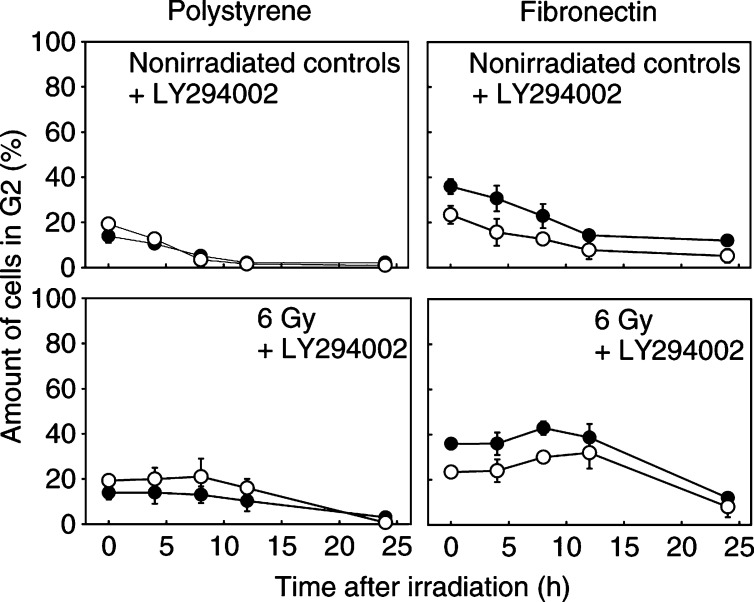
Cell cycle alterations by the PI3-K-specific inhibitor LY294002 (LY) were analysed in the presence or absence of FN using nonirradiated A549 and CCD32 controls in comparison with irradiated cells. Irradiation was delivered following an 18-h incubation with 50 *μ*M of LY294002 in serum-free medium. Each data point represents the mean±s.d. of three independent experiments. LY294002 led to a strong decrease in G2 phase cells on polystyrene, which was partly prevented by FN. Radiation-dependent accumulation of cells in the G2 phase was completely prevented on polystyrene and markedly decreased on FN in comparison with irradiated cells not exposed to LY294002 as shown in Figure 4.

With regard to the convergence of integrin and growth factor signalling cascades, we analysed whether the detected PI3-K-dependent and -independent changes in ILK, PKB*α*/Akt activity and GSK-3*β* phosphorylation (see Figure 2D) could be responsible for alterations in cell cycle progression, cyclin D1 expression and pRb phosphorylation (Figure 6

and Figure 7

**Figure 7:**
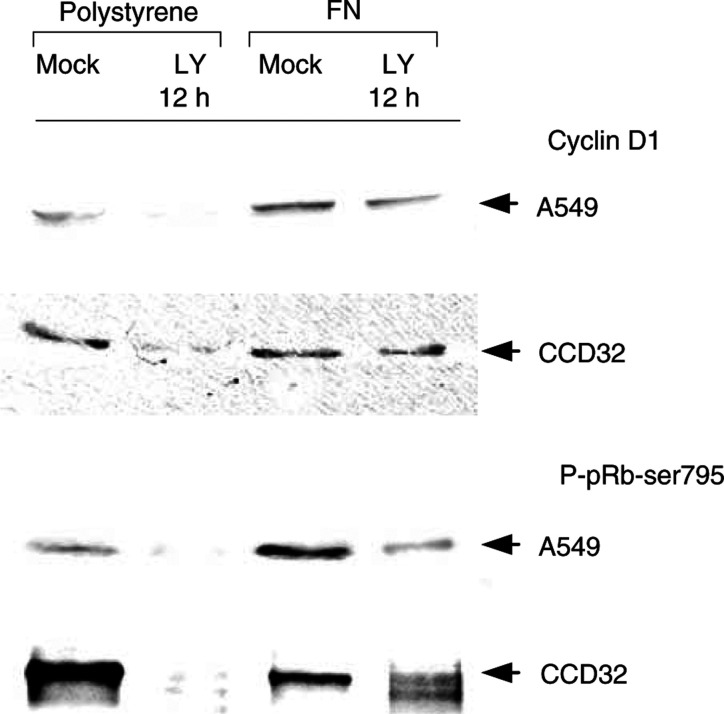
Cyclin D1 expression and pRb phosphorylation were investigated after a 12-h LY294002 (50 *μ*M) incubation in A549 and CCD32 cells grown on polystyrene or FN in comparison with untreated controls (mock). Correlating the decreases of cyclin D1 expression and pRb phosphorylation with the DNA analysis shown in Figure 6 supported the important role of PI3-K-dependent growth factor pathways for the regulation of cell division and associated cell cycle proteins at FN presence and absence. Most interestingly, cells attached to FN did not demonstrate a downregulation of cyclin D1 expression or pRb phosphorylation, which indicates a strong influence of ECM on the cell cycle regulation via ILK-GSK-3*β*.

). LY294002 exposure showed marked decreases in the G2 cell fraction of A549 cells on polystyrene (2.2%) or FN (12%) and of CCD32 cells on polystyrene (1%) or FN (5.2%) compared to noninhibited controls (see Figure 4). The radiation-dependent G2 cell accumulation was completely impaired in cells grown on polystyrene and decreased in cells grown on FN (Figure 6). Concomitantly to changes detected in G2, cells in the G0/G1 and more prominent in the S phase increased. In parallel, cyclin D1 and phospho-pRb expression were downregulated after a 12-h incubation with LY294002 on polystyrene but, most interestingly, not on FN (Figure 7).

## DISCUSSION

**Figure 8:**
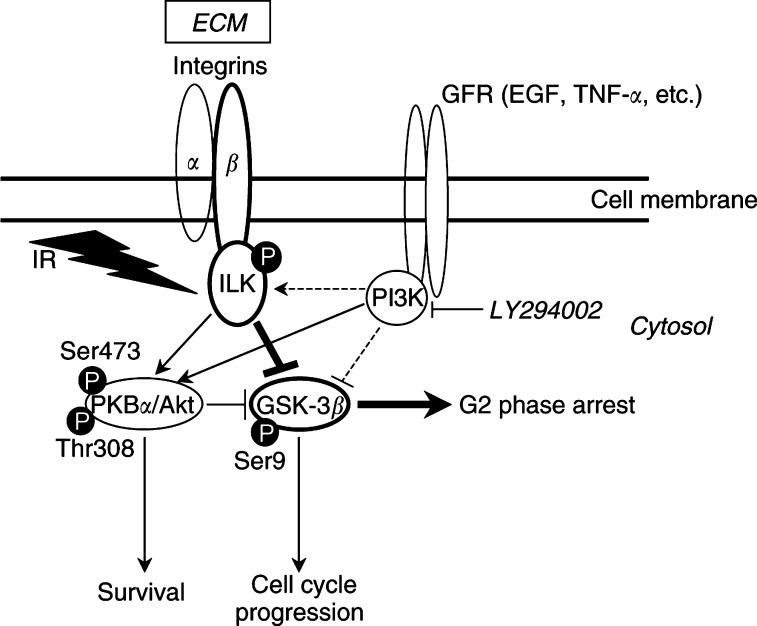
Schematic diagram of how integrin-linked kinase (ILK) and glycogen synthase kinase-3*β* (GSK-3*β*) might be involved in the acute radiation response with regard to cell survival, cell cycle progression and initiation of G2 phase arrest. Binding of cells to extracellular matrix (ECM) components via *β*1-integrins stimulates ILK and downstream targets protein kinase B*α*/Akt (PKB*α*/Akt) (phosphorylation at amino-acid residues Ser473 and Thr308) and GSK-3*β* (phosphorylation at amino-acid residue Ser9). These events suppress apoptosis and promote survival by inhibiting Bad and caspase-9 and cell cycle transition by blocking proteolysis of cyclin D1. Facilitating growth factor binding to growth factor receptors (GFR) activates similar pathways downstream of the central regulator phosphatidylinositol-3 kinase (PI3-K). In bold letters, arrows and circles, we suggest and thereby support the hypothesis of direct ILK phosphorylation of GSK-3*β* when PI3-K is inhibited and cells are attached to ECM. Irradiation (IR) is able to activate this pathway, which then does not stimulate proliferation but rather blocks cells in the G2 phase possibly allowing damage repair.

Physiologically normal and malignant cells are imbedded into an ECM, which is a requirement for cell proliferation and survival for a wide variety of cell types (Meredith *et al*, 1993; Frisch and Francis, 1994). Furthermore, the ECM might have a great impact on the cellular responsiveness to external stimuli. Published *in vitro* and *in vivo* findings showed the importance of the extracellular environment on drug (Sethi *et al*, 1999; Hazlehurst *et al*, 2000) and radiation sensitivity (Vlodavsky *et al*, 1980; Cordes *et al*, 2002; Cordes and Meineke, in press; Cordes *et al*, in press), conferring resistance to DNA-damaging agents. Since integrin and growth factor receptors are colocalised at the source of integrin signalling, which are called focal adhesions, convergence and mutual modification between these networks are highly likely to occur. This crosstalk could result in differentiated regulative schemes of cellular functions such as cell growth and survival and, thus, may be central to our understanding of resistance-mediating mechanisms. These mechanisms are not only important for single tumour or normal cells, but are possibly of outstanding interest for a growing tumour that consists of organ-specific, already transformed epithelial cells and the adjacent stroma containing normal cells such as fibroblasts, endothelial cells and leucocytes (Seljedid *et al*, 1999). Focusing on tumour and fibroblastic cells of, for example, a human lung cancer, the cells correspond intensely via cell–cell contact and soluble signalling molecules that modulate the composition of the extracellular environment. These interactions have been reported to be able to support strongly the growth of the tumour (Lukashev and Werb, 1998; Kunz-Schughart and Knuechel, 2002). On the one hand, every single cell responds on its own upon treatment with a cytotoxic agent like ionising radiation, but, on the other, there occur multiple interactions between these two neighbours, which might modulate the cellular radiation response in terms of improved radioresistance. In this study, our *in vitro* results show, for the first time, a participation of the widespread *β*1-integrin subunit-associated protein kinases, ILK, PKB*α*/Akt, GSK-3*β* within the radiation response of a human lung cancer cell line as well as normal human lung primary fibroblasts. These data indicate that these specific kinases are likely to be involved in improved radioresistance at ECM presence, in ECM-modulated cell cycle progression and the expression of cyclin D1 and pRb (Figure 8

).

The major findings of this study include, firstly, a significantly improved clonogenic survival of A549 and CCD32 cells after irradiation in the presence of FN or LA in contrast to polystyrene, BSA or poly-L; secondly, ILK and PKB*α*/Akt stimulation and increased GSK-3*β* phosphorylation by ionising radiation in a matrix-dependent manner; thirdly, a PI3-K-independent ILK stimulation and GSK-3*β* inhibition by irradiation at FN presence in combination with undisturbed cyclin D1 expression and pRb phosphorylation; fourthly, a prolongation and increase of the radiation-induced G2 phase arrest by FN which could be correlated with the expression patterns of cell cycle proteins and which could be impaired by PI3-K inhibition in a matrix-dependent manner. Additionally, basal protein kinase activities are shown to be markedly elevated when cells had FN contact.

Cell survival and cell growth regulated by growth factor signalling (Moustakas *et al*, 2002; Neve *et al*, 2002) have been shown by Wu *et al* (1998) to be also substantially affected by ILK signalling upon ligand binding to *β*1-integrin receptors. The ILK signalling cascade seems to be involved in tumour growth, tumorigenesis and in the development of a metastatic phenotype. Recently, White *et al* (2001) provided direct evidence for the oncogenic potential of ILK in the induction of mammary gland hyperplasia and formation of tumours in transgenic mice *in vivo*. The observed ILK overexpression was accompanied by a constitutive phosphorylation of the ILK downstream targets PKB*α*/Akt and GSK-3*β*. To date, investigating these molecular factors under cell treatment with cytotoxic agents such as ionising radiation or drugs has only uncovered PKB*α*/Akt but not ILK or GSK-3*β* involvement. PKB*α*/Akt affects cellular radiosensitivity (Akimoto *et al*, 2001) in a PI3-K- (Enns *et al*, 1999; Brognard *et al*, 2001) or protein kinase C-dependent manner (Tenzer *et al*, 2001) leading to changes in GSK-3*α* phosphorylation and alterations of cellular radiosensitivity. Concerning DNA damage caused by chemical agents such as camptothecin, Watcharasit *et al* (2002) were able to show GSK-3*β* participation within the cellular response of DNA repair. However, changes in basal kinase activities because of cell–ECM contact have not been taken into account. As shown here, cell growth on FN, which characterises a more physiologic experimental condition, activated the *β*1-integrin pathway *per se* in a cell type-independent manner. With regard to the improved clonogenic survival, we hypothesise that this basal activation provides an optimised physiologic status for the cell to counteract external stimuli. The radiation-mediated stimulation (ILK, PKB*α*/Akt) and inhibition (GSK-3*β*) indicates the involvement of the tested protein kinases in the acute radiation response mechanisms of A549 and CCD32 cells.

Elucidating the process of cell cycle transition that is markedly influenced by irradiation in that cells respond by delaying the cell cycle at checkpoints in the G1- or G2-phase (Lane, 1992; Gadbois *et al*, 1997; Dimitrijevic-Bussod *et al*, 1999), additionally showed a regulative role for cell adhesion to ECM (Shin *et al*, 1975; Hannigan *et al*, 1996; Kang and Krauss, 1996; Zhu *et al*, 1996). These checkpoint delays are believed to provide the cell with more time to repair the damage-preserving genome integrity in daughter cells before they continue the division cycle (Little and Nagasawa, 1985; Lane, 1992). Since there is a huge gap in our knowledge concerning the effects of the ECM on radiation-dependent cell cycle arrest, the presented data give first evidence that the ECM protein FN enables tumour and, especially, normal cells to delay for a longer period of time in the G2 phase possibly promoting DNA repair and thus survival. However, comparing basal and radiation-altered cell cycle distributions, the proportional differences of the G2 cell amounts on polystyrene or FN suggest a pronounced FN-related change in the cellular responsiveness of the lung fibroblasts rather than the lung cancer cells to irradiation. This effect resulted in an increase of survival that is likely to be in part because of an optimised DNA repair machinery. In contrast, A549 cells possibly posses specific genomic mutations that provide a survival advantage independent of an FN-mediated overproportional G2/DNA repair phase.

While the mitogenesis signal involves the activation of the ras-raf-mitogen-activated protein kinase (MAPK) and PI3-K signalling pathways (Chen *et al*, 1994; Dedhar and Hannigan, 1997), the pathways participating in integrin-mediated regulation of survival and differentiation are not well characterised but have also implicated PI3-K, MAPK and PKB/Akt (Khwaja *et al*, 1997). Especially, the cell cycle seems to be proceeded by a PKB/Akt-independent mechanism involving GSK-3, *β*-catenin or AP-1 transcription factor (Radeva *et al*, 1997; Troussard *et al*, 1999). Using the specific PI3-K inhibitors LY294002 and wortmannin, we were able to show that both radiation- and adhesion-mediated activation of the ILK-PKB*α*/Akt-GSK-3*β* pathway were partly PI3-K-dependent. In fact, we provide evidence for a radiation-inducible, PI3-K-independent ILK-GSK-3*β* signalling pathway in the case of cell–FN contact. With regard to cyclin D1 expression and pRb phosphorylation at PI3-K inhibition, the protein patterns showed only slight changes when cells were plated on FN. In contrast, the PKB*α*/Akt cascades indicate that not they are susceptible to either ILK or radiation without PI3-K support. These findings strongly suggest that only the combination of cell anchorage to ECM plus growth factors is able to modulate cellular responsiveness to ionising radiation most effectively and, thus, suggesting a tight convergence between these pathways already taking place upstream at the cytoplasmic face of the cell membrane.

The results of this study identify ILK, PKB*α*/Akt and GSK-3*β* within the critical *β*1-integrin pathway as important molecular factors to regulate individual cellular radiosensitivity in the presence of an ECM. We could uncover a novel radiation-inducible, PI3-K-independent and membrane-located pathway via ILK and GSK-3*β*. Basal cell cycle transition as well as the radiation-induced G2 arrest was intensively altered by FN in a PI3-K-dependent manner. On the basis of great similarity of the results generated in the human lung cancer cells A549 and the normal human lung fibroblasts CCD32, most of the cell adhesion-transduced resistance mechanisms seem to work independent of the genetic and differentiation status of the cell. The detailed identification of the molecular mechanisms will possibly provide considerable insight into the understanding of cell adhesion-mediated drug and radioresistance, cell–ECM-interactions and tumour growth with respect to the modulation of multiple cellular network convergence by the microenvironment.

## References

[bib1] Akimoto T, Nonaka T, Ishikawa H, Sakurai H, Saitoh JI, Takahashi T, Mitsuhashi N (2001) Genistein, a tyrosine kinase inhibitor, enhanced radiosensitivity in human esophageal cancer cell lines *in vitro* : possible involvement of inhibition of survival signal transduction pathways. Int J Radiat Oncol Biol Phys 50: 195–201 11316564 10.1016/s0360-3016(00)01560-1

[bib2] Argraves WS, Suzuki S, Arai H, Thompson K, Pierschbacher MD, Ruoslathi E (1987) Amino acid sequence of the human fibronectin receptor. J Cell Biol 105: 1183–1190 2958481 10.1083/jcb.105.3.1183 PMC2114793

[bib3] Brognard J, Clark AS, Ni Y, Dennis PA (2001) Akt/protein kinase B is constitutively active in non-small cell lung cancer cells and promotes cellular survival and resistance to chemotherapy and radiation. Cancer Res 61: 3986–3997 11358816

[bib4] Chen Q, Kinch MS, Lin TH, Burridge K, Juliano RL (1994) Integrin-mediated cell adhesion activates mitogen-activated protein kinases. J Biol Chem 269: 26602–26605 7929388

[bib5] Cordes N, Blaese MA, Meineke V, van Beuningen D (2002) Ionizing radiation induces up-regulation of functional *β*1-integrin in lung tumour cell lines *in vitro*. Int J Radiat Biol 78: 347–357 12020426 10.1080/09553000110117340

[bib6] Cordes N, Blaese MA, Plasswilm L, Rodemann HP, van Beuningen D (2002) Fibronectin and laminin increase resistance to ionising radiation and the cytotoxic drug Ukrain® in human tumour and normal cells *in vitro*. Int J Radiat Biol, in press 10.1080/09553000310001610240 14703944

[bib7] Cordes N, Meineke V (2002) Cell adhesion-mediated radioresistance (CAM-RR): extracellular matrix-dependent improvement of cell survival in human tumor and normal cells *in vitro*. Strahlenther Onkol, in press 10.1007/s00066-003-1074-4 12740661

[bib8] Cross DA, Alessi DR, Cohen P, Andjelkovich M, Hemmings BA (1995) Inhibition of glycogen synthase kinase 3 by insulin mediated by protein kinase B. Nature 378: 785–789 8524413 10.1038/378785a0

[bib9] Damiano JS, Cress AE, Hazlehurst LA, Shtil AA, Dalton WS (1999) Cell adhesion mediated drug resistance (CAM-DR): role of integrins and resistance to apoptosis in human myeloma cell lines. Blood 93: 1658–1667 10029595 PMC5550098

[bib10] Datta SR, Dudek H, Tao X, Masters S, Fu H, Gotoh Y, Greenberg ME (1997) Akt phosphorylation of BAD couples survival signals to an cell-intrinsic death machinery. Cell 91: 231–241 9346240 10.1016/s0092-8674(00)80405-5

[bib11] Dedhar S, Hannigan GE (1997) Integrin cytoplasmic interactions and bidirectional transmembrane signalling. Curr Opin Cell Biol 8: 657–669 10.1016/s0955-0674(96)80107-4 8939656

[bib12] Delcommenne M, Tan C, Gray V, Ruel L, Woodgett J, Dedhar S (1998) Phosphoinositide-3-OH kinase-dependent regulation of glycogen synthase kinase 3 and protein kinase B/Akt by the integrin-linked kinase. Proc Natl Acad Sci USA 95: 11211–11216 9736715 10.1073/pnas.95.19.11211 PMC21621

[bib13] Diehl JA, Cheng M, Roussel MF, Sherr CJ (1998) Glycogen synthase kinase-3beta regulates cyclin D1 proteolysis and subcellular localization. Genes Dev 12: 3499–3511 9832503 10.1101/gad.12.22.3499 PMC317244

[bib14] Dimitrijevic-Bussod M, Balzaretti-Maggi VS, Gadbois DM (1999) Extracellular matrix and radiation G1 cell cycle arrest in human fibroblasts. Cancer Res 59: 4843–4847 10519394

[bib15] Enns L, Murray D, Mirzayans R (1999) Effects of the protein kinase inhibitors wortmannin and KN62 on cellular radiosensitivity and radiation-activated S phase and G1/S checkpoints in normal human fibroblasts. Br J Cancer 81: 959–965 10576651 10.1038/sj.bjc.6690793 PMC2362948

[bib16] Frisch SM, Francis H (1994) Disruption of epithelial cell–matrix interactions induces apoptosis. J Cell Biol 124: 619–626 8106557 10.1083/jcb.124.4.619 PMC2119917

[bib17] Gadbois DM, Bradbury EM, Lehnert BE (1997) Control of radiation-induced G1 arrest by cell–substratum interactions. Cancer Res 57: 1151–1156 9067286

[bib18] Giancotti FG, Ruoslahti E (1999) Integrin signaling. Science 285: 1028–1032 10446041 10.1126/science.285.5430.1028

[bib19] Hannigan GE, Leung-Hagesteijn C, Fitz-Gibbon L, Coppolino MG, Radeva G, Filmus J, Bell JC, Dedhar S (1996) Regulation of cell adhesion and anchorage-dependent growth by a new *β*1-integrin-linked protein kinase. Nature 379: 91–96 8538749 10.1038/379091a0

[bib20] Hazlehurst LA, Damiano JS, Buyuksal I, Pledger WJ, Dalton WS (2000) Adhesion to fibronectin via *β*1-integrins regulates p27kip1 levels and contributes to cell adhesion mediated drug resistance (CAM-DR). Oncogene 19: 4319–4327 10980607 10.1038/sj.onc.1203782

[bib21] Hoeflich KP, Luo J, Rubie EA, Tsao MS, Jin O, Woodgett JR (2000) Requirement for glycogen synthase kinase-3beta in cell survival and NK-*κ*B activation. Nature 406: 86–90 10894547 10.1038/35017574

[bib22] Hynes RO (1992) Integrins: versatility, modulation, and signaling in cell adhesion. Cell 69: 11–25 1555235 10.1016/0092-8674(92)90115-s

[bib23] Kang JS, Krauss RS (1996) Ras induces anchorage-independent growth by subverting multiple adhesion-regulated cell cycle events. Mol Cell Biol 16: 3370–3380 8668152 10.1128/mcb.16.7.3370 PMC231331

[bib24] Khwaja A, Rodriguez-Viciana P, Wennström S, Warne HP, Downward J (1997) Matrix adhesion and Ras transformation both activate a phosphoinositide 3-OH kinase and protein kinase B/Akt cellular survival pathway. EMBO J 16: 2783–2793 9184223 10.1093/emboj/16.10.2783 PMC1169887

[bib25] Kunz-Schughart LA, Knuechel R (2002) Tumor-associated fibroblasts (part I): active stromal participants in tumor development and progression? Histol Histopathol 17: 599–621 11962761 10.14670/HH-17.599

[bib26] Lane DP (1992) p53: guardian of the genome. Nature 358: 15–16 1614522 10.1038/358015a0

[bib27] Little JB, Nagasawa H (1985) Effect of confluent holding on potentially lethal damage repair, cell cycle progression, and chromosomal aberrations in human normal and ataxia-telangiectasia fibroblasts. Radiat Res 101: 81–93 3969444

[bib28] Lukashev ME, Werb Z (1998) ECM signalling: orchestrating cell behaviour and misbehaviour. Trends Cell Biol 8: 437–441 9854310 10.1016/s0962-8924(98)01362-2

[bib29] Meredith Jr JE, Fazeli B, Schwartz MA (1993) The extracellular matrix as a cell survival factor. Mol Biol Cell 4: 953–961 8257797 10.1091/mbc.4.9.953 PMC275725

[bib30] Moustakas A, Pardali K, Gaal A, Heldin CH (2002) Mechanisms of TGF-beta signaling in regulation of cell growth and differentiation. Immunol Lett 82: 85–91 12008039 10.1016/s0165-2478(02)00023-8

[bib31] Neve RM, Holbro T, Hynes NE (2002) Distinct roles for phosphoinositide 3-kinase, mitogen-activated protein kinase and p38 MAPK in mediating cell cycle progression of breast cancer cells. Oncogene 21: 4567–4576 12085235 10.1038/sj.onc.1205555

[bib32] Pawson T, Saxton TM (1999) Signaling networks –do all roads lead to the same genes? Cell 97: 675–678 10380918 10.1016/s0092-8674(00)80779-5

[bib33] Radeva G, Petrocelli T, Behrend E, Leung-Hagesteijn C, Filmus J, Slingerland J, Dedhar S (1997) Overexpression of the integrin-linked kinase promotes anchorage-independent cell cycle progression. J Biol Chem 272: 13937–13944 9153256 10.1074/jbc.272.21.13937

[bib34] Rose RW, O'Hara MO, Williamson SK, Grant DS (1999) The role of laminin-1 in the modulation of radiation damage in endothelial cells and differentiation. Radiat Res 152: 14–28 10381837

[bib35] Schaller MD, Parsons JT (1994) Focal adhesion kinase and associated proteins. Curr Opin Cell Biol 6: 705–710 7833050 10.1016/0955-0674(94)90097-3

[bib36] Schwartz MA, Baron V (1999) Interactions between mitogenic stimuli or, a thousand and one connections. Curr Opin Cell Biol 11: 197–202 10209147 10.1016/s0955-0674(99)80026-x

[bib37] Schwartz MA, Ginsberg MH (2002) Networks and crosstalk: integrin signalling spreads. Nat Cell Biol 4: E65–E68 11944032 10.1038/ncb0402-e65

[bib38] Seljedid R, Jozefowski S, Sveinbjornsson (1999) Tumor stroma. Anticancer Res 19: 4809–4822 10697594

[bib39] Sethi T, Rintoul RC, Moore SM, MacKinnon AC, Salter D, Choo C, Chilvers ER, Dransfield I, Donnelly SC, Strieter R, Haslett C (1999) Extracellular matrix proteins protect small cell lung cancer cells against apoptosis: a mechanism for small cell lung cancer growth and drug resistance *in vivo*. Nat Med 5: 662–668 10371505 10.1038/9511

[bib40] Shin S-I, Freedman VH, Risser R, Pollack R (1975) Tumorigenicity of virus-transformed cells in nude mice is correlated specifically with anchorage-independent growth *in vitro*. Proc Natl Acad Sci USA 72: 4435–4439 172908 10.1073/pnas.72.11.4435 PMC388736

[bib41] Tenzer A, Zingg D, Rocha S, Hemmings B, Fabbro D, Glanzmann C, Schubiger PA, Bodis S, Pruschy M (2001) The phosphatidylinositide 3 ′-kinase/Akt survival pathway is a target for the anticancer and radiosensitizing agent PKC412, an inhibitor of protein kinase C. Cancer Res 61: 8203–8210 11719451

[bib42] Tian B, Lessan K, Kahm J, Kleidon J, Henke C (2002) *β*1 integrin regulates fibroblast viability during collagen matrix contraction through a phosphatidylinositol 3-kinase/Akt/protein kinase B signaling pathway. J Biol Chem 27: 24667–24675 10.1074/jbc.M203565200 11986332

[bib43] Troussard AA, Tan C, Yoganathan TN, Dedhar S (1999) Cell–extracellular matrix interactions stimulate the AP-1 transcription factor in an integrin-linked kinase- and glycogen synthase kinase 3-dependent manner. Mol Cell Biol 19: 7420–7427 10523630 10.1128/mcb.19.11.7420 PMC84735

[bib44] Vlodavsky I, Lui GM, Gospodarowicz D (1980) Morphological appearance, growth behaviour and migratory activity of human tumor cells maintained on extracellular matrix *vs* plastic. Cell 19: 607–616 6965887 10.1016/s0092-8674(80)80037-7

[bib45] Watcharasit P, Bijur GN, Zmijewski JW, Song L, Zmijewska A, Chen X, Johnson GVW, Jope RS (2002) Direct, activating interactions between glycogen synthase kinase-3*β* and p53 after DNA damage. Proc Natl Acad Sci USA 99: 7951–7955 12048243 10.1073/pnas.122062299 PMC123001

[bib46] White DE, Cardiff RD, Dedhar S, Muller WJ (2001) Mammary epithelial-specific expression of the integrin-linked kinase (ILK) results in the induction of mammary gland hyperplasias and tumors in transgenic mice. Oncogene 20: 7064–7072 11704830 10.1038/sj.onc.1204910

[bib47] Wu C, Keightley SY, Leung-Hagesteijn C, Radeva G, Coppolino M, Goicoechea S, McDonald JA, Dedhar S (1998) Integrin-linked kinase regulates fibronectin matrix assembly, E-cadherin expression and tumorigenicity. J Biol Chem 273: 528–536 9417112 10.1074/jbc.273.1.528

[bib48] Wu RC, Schönthal AH (1997) Activation of p53–p21waf1 pathway in response to disruption of cell–matrix interactions. J Biol Chem 272: 29091–29098 9360984 10.1074/jbc.272.46.29091

[bib49] Yamada KM, Even-Ram S (2002) Integrin regulation of growth factor receptors. Nat Cell Biol 4: E75–E76 11944037 10.1038/ncb0402-e75

[bib50] Zhu X, Ohtsubo M, Bohmer RM, Roberts JM, Assoian RK (1996) Adhesion-dependent cell cycle progression linked to the expression of cyclin D1, activation of cyclin E-cdk2, and phosphorylation of the retinoblastoma protein. J Cell Biol 133: 391–403 8609171 10.1083/jcb.133.2.391 PMC2120799

